# Dynamic Profiling of Circulating Tumor Cells and *MYC/PTEN* Alterations in Prostate Cancer Patients Undergoing Radical Prostatectomy in Amazon Population

**DOI:** 10.1002/mc.70104

**Published:** 2026-03-06

**Authors:** Juliana Ramos Chaves, Rommel Mario Rodriguez Burbano, Amanda de Nazaré Cohen‐Paes, Diego Di Felipe Ávila Alcântara, Ana Paula Borges de Souza, Maria Janete Nahum Gomes, Sérgio Augusto Antunes Ramos, André Salim Khayat

**Affiliations:** ^1^ Laboratório de Medicina Molecular Universidade Federal do Pará Belém Pará Brazil; ^2^ Serviço de Urologia Hospital Ophir Loyola Belém Pará Brazil; ^3^ Núcleo de Pesquisas em Oncologia, Hospital Universitário João de Barros Barreto R. dos Mundurucus, 4487 ‐ Guamá Belém Pará Brazil

**Keywords:** biomarker, circulating tumor cells, prostate cancer, PTEN/MYC, surgery

## Abstract

Prostate cancer (PCa) is the second most frequently diagnosed malignancy among men and the sixth leading cause of cancer‐related mortality worldwide. Radical prostatectomy remains the standard treatment for localized disease; however, approximately one‐third of patients develop biochemical recurrence within 10 years. Although prostate‐specific antigen (PSA) is the primary biomarker used for diagnosis and monitoring, additional molecular markers such as *PTEN* loss and *MYC* amplification have been investigated to improve prognostic stratification. Liquid biopsy has emerged as a promising tool in oncology, yet the clinical significance of circulating tumor cells (CTCs) in early‐stage PCa remains uncertain. This prospective, single‐center study included 50 patients with localized prostate cancer from the Brazilian Amazon region who underwent radical prostatectomy in 2023. Peripheral blood samples were collected preoperatively and postoperatively for CTC enumeration and molecular characterization using Cytelligen SET‐iFISH and the CellSearch system. *PTEN* and *MYC* status were evaluated to further characterize the detected CTC population. CTCs were detected in all patients prior to surgery, with a significant reduction observed after radical prostatectomy (*p* < 0.001); 88% of patients had no detectable CTCs postoperatively. *PTEN* loss was identified in 12% of patients, all of whom presented with ISUP grade ≥ 2 disease. *MYC* amplification was rare, observed in only one patient with Gleason score 7 and concomitant *PTEN* deletion. These findings suggest that perioperative CTC detection may provide clinically relevant prognostic information in localized PCa. *PTEN* loss appears to represent an early molecular alteration associated with adverse pathological features. Longer follow‐up is needed to determine the predictive value of these biomarkers for recurrence and long‐term outcomes.

**Abbreviations**
ADT: androgen deprivation therapy
CTCs: circulating tumor cells
ctDNA: circulating tumor DNA
DAPI: 4′,6‐diamidino‐2‐phenylindole
HOL: Hospital Ophir Loyola
iFISH: interphase fluorescent *in situ* hybridization
ISUP: International Society of Urological Pathology
LB: liquid biopsy
miRNAs: microRNAs
PCa: prostate cancer
PSA: prostate specific Antigen
SET: cytelligen kit for subtraction enrichment

## 1 Introduction

Prostate cancer (PCa) is the second most common malignancy among men and the sixth leading cause of cancer‐related death worldwide [1]. This disease exhibits considerable biological heterogeneity and geographic variability, influenced by differences in screening, treatment accessibility, environmental exposures, and genetic background [2, 3]. The classic method of histopathological classification of PCa is done through the Gleason scoring system, used to assess the degree of aggressiveness of prostate adenocarcinoma. It is based on the analysis of glandular architectural patterns observed under a microscope, classified from 1 to 5 according to the level of cell differentiation [4]. The sum of the two predominant patterns, known as the Gleason Score, reflects the biological behavior of the tumor, with higher values indicating greater structural disorganization and aggressive potential.

With the aim of improving prognostic accuracy and reducing the interpretative heterogeneity of the original system, the International Society of Urologic Pathology (ISUP) proposed a new stratification model called the ISUP Grade Group. This system reorganizes the possible combinations of the Gleason Score into five graded groups, ranging from Grade 1 (well‐differentiated tumors, Gleason ≤ 6) to Grade 5 (poorly differentiated tumors, Gleason 9–10). The ISUP classification has become widely adopted because it offers greater clinical clarity, better correlation with prognostic outcomes, and greater consistency among pathologists [5].

Treatment strategies for PCa are based on the initial staging of the disease. For localized disease, primary options include local therapy, radical prostatectomy, or radiotherapy, with or without androgen deprivation therapy (ADT), while active surveillance may be considered for carefully selected patients with low‐risk or favorable intermediate‐risk disease. Therapeutic decisions incorporate tumor staging, Gleason/ISUP grading, prostate specific antigen (PSA) levels, patient comorbidities, age, and individual preferences [6].

Radical prostatectomy remains the standard surgical treatment; however, approximately one‐third of patients experience recurrence within 10 years [7]. Postoperative PSA levels are expected to drop to undetectable values, indicating successful removal of malignant and benign prostate tissue [8]. PSA monitoring remains a key strategy for detecting biochemical recurrence. Although widely used for screening and prognostic evaluation, PSA has limited sensitivity and specificity, motivating the search for complementary biomarkers [9, 10]. In this context, a range of genomic biomarkers has been implicated in the initiation and progression of PCa.

The tumor suppressor gene *PTEN* plays a central role in regulating cell growth, differentiation, and survival across various cancers, including PCa. Loss of *PTEN* function promotes activation of the *PI3K*/*AKT* signaling pathway, enhancing proliferation, migration, and cell survival [11]. *PTEN* loss is significantly associated with disease stage, being most prevalent in metastatic castration‐resistant PCa [12]. Studies estimate *PTEN* loss occurs in 15%–20% of primary tumors, increasing to 40%–60% in advanced disease [13, 14, 15].

The *MYC* oncogene is frequently amplified in PCa and linked to tumor progression and chromatin remodeling via interaction with the CTCF protein, impacting androgen receptor–regulated gene transcription [16, 17, 18]. *MYC* alterations are associated with aggressive disease and poor prognosis.

Given the increasing importance of molecular profiling in PCa, liquid biopsy (LB) has emerged as a promising, minimally invasive tool for detecting biomarkers, such as circulating tumor cells (CTCs), circulating tumor DNA (ctDNA), and microRNAs (miRNAs) in cancer patients [19, 20]. LB offers advantages over traditional tissue biopsies, particularly in early detection, longitudinal monitoring, and characterization of tumor heterogeneity [21].

In PCa, CTCs have been widely studied as they mirror the genetic alterations of primary tumors and are associated with metastatic initiation [22]. A study of 155 treatment‐naïve patients with localized PCa found CTCs in 54% of cases, correlating with higher Gleason scores, risk groups, and clinical significance [23]. Detection rates are substantially higher in metastatic disease, though sensitivity remains a limitation [24, 25].

The identification of CTCs using the CellSearch® system (Menarini Silicon Biosystems, San Diego, CA, USA) has been validated in multiple studies as both a prognostic marker and an early indicator of treatment response in PCa [26]. The presence of > 5 CTCs per 7.5 mL blood has been associated with shorter overall survival, outperforming PSA in prognostic prediction [27, 28, 29, 30].

Despite the growing literature on the prognostic significance of CTCs across multiple cancers, robust clinical validation of their role in diagnosis and therapeutic monitoring of PCa remains limited. This highlights the need for further studies to establish their broader clinical utility.

This study aims to evaluate the prognostic potential of CTC enumeration in patients with localized PCa before and after radical prostatectomy using LB. Additionally, we analyze *MYC* and *PTEN* status to molecularly characterize the CTC population and investigate their correlation with clinical outcomes.

## 2 Material and Methods

### 2.1 Experimental Model and Study Participant Details

This prospective, observational, single‐center study included 50 male patients diagnosed with prostate adenocarcinoma who underwent radical prostatectomy at Ophir Loyola Hospital (Pará, Brazil) during 2023. Inclusion criteria comprised histopathologically confirmed PCa, age over 18 years, and no prior treatment. Exclusion criteria included evidence of metastasis, neoadjuvant hormonal therapy, or refusal to provide consent.

For the minimum sample size calculation, the following sample size formula was used:

$$\frac{\frac{z^{2}\times p(1-p)}{e^{2}}}{1+\left(\frac{z^{2}\times p(1-p)}{e^{2}N}\right)}.$$

Based on a 95% confidence level and a 5% margin of error, the sample comprised 50 participants/respondents.

Venous blood samples (7 mL Ethylenediaminetetraacetic acid [EDTA]) were collected from participants on the day before surgery and 30 days postoperatively, serving as LB material for biomarker analysis. Clinical and pathological data were collected prospectively from the patients' medical records, including staging, histopathological results, Gleason score (not analyzed in this work), ISUP grade group, and pre and postoperative PSA levels.

The present study was conducted in accordance with ethical standards established by the Nuremberg Code and the Declaration of Helsinki, which underpin Resolution CNS No. 196/96 for the creation and development of research involving human subjects. After evaluation and approval by the Research Ethics Committee of Hospital Ophir Loyola (HOL) (No. 64401316.5.0000.5634), all participants signed a Free and Informed Consent Form and received guidance regarding the benefits, risks, and the data collection procedures involved.

### 2.2 Inclusion and Exclusion Criteria

The inclusion criteria were: (1) histologically confirmed diagnosis of prostate adenocarcinoma; (2) males (18+ years); (3) indication for radical prostatectomy as the initial therapy; and (4) patients who had not yet undergone any form of treatment.

The exclusion criteria applied were: (1) confirmed metastatic disease by bone scintigraphy; (2) receipt of any hormonal therapy for PCa; and (3) refusal to sign the informed consent form.

### 2.3 Detection and Counting of CTCs

The present experiment was conducted following the instructions of the Cytelligen kit for Subtraction Enrichment (SET) and subsequent detection via fluorescent in situ hybridization for CTCs (SET‐iFISH) (Cytelligen, San Diego, CA, USA).

Blood samples from the study participants were collected in 7.5 mL tubes containing acid citrate dextrose anticoagulant (Becton Dickinson, Franklin Lakes, NJ, USA). Hemolytic removal of erythrocytes was performed using Cytelligen's cell separation matrix (Cytelligen), following the manufacturer's instructions. CTC counting was carried out using the CellSearch system.

The blood samples were placed into 10 mL CellSave Vacutainer tubes (Becton Dickinson, Franklin Lakes, NJ, USA) containing EDTA and cell fixative. The samples were kept at room temperature for up to 72 h before processing with an automated system (CellPrep) and the CellSearch Epithelial Cell Kit. Cells expressing epithelial cell adhesion molecules were captured with antibody‐coated iron particles, and nuclei were stained with 4',6‐diamidino‐2‐phenylindole (DAPI).

Monoclonal antibodies conjugated to fluorescent dyes against leukocytes (CD45) and cytokeratins CK‐8,‐18,‐19 were used to distinguish epithelial cells from leukocytes. Additional identification and counting of CTCs were performed using the CellSpotterAnalyzer, an automated fluorescence microscopy system that generates computer‐reconstructed cellular images. CTCs were defined as nucleated cells with positive CK staining and negative CD45 staining.

### 2.4 iFISH

The iFISH technique was used to visualize CTCs and detect deletions, duplications, and chromosomal translocations. Dried CTC‐coated slides were washed and incubated with PBS buffer (3′), then hybridized with Vysis probes for *PTEN* and *MYC* (SpectrumOrange) for 4 h. Samples were next incubated with fluorochrome‐conjugated monoclonal antibodies against CD31 and CD45 (20′, dark), washed, stained with DAPI, and analyzed by automated scanning. CTCs identified by SET‐iFISH showed the profile: DAPI+ (blue), FISH+ (aneuploid allele), CD31− (green), and CD45− (red).

Lamellae were placed on slides, denatured at 80°C for 10 min, and hybridized at 37°C overnight. After removing lamellae, slides were washed sequentially: twice in 0.1 x SSC/1.5 M urea at 45°C (20 min), in 2 x SSC at 45°C (10 min), and in 2 x SSC/0.1% NP‐40 at 45°C (10 min). Finally, slides were washed in 2 x SSC at room temperature (5 min), air‐dried, and counterstained with 10 µL DAPI/Antifade (DAPI in Fluorguard, 0.5 µg/mL, Insitus, Albuquerque, NM).

The choice of these molecular markers was based on the fact that they are key genes in the control of proliferative and cell survival pathways, driven especially by *MYC*, and negatively regulated by *PTEN*, as is the case with the *PI3K*/*AKT*/*mTOR* pathway.

### 2.5 Quantification and Statistical Analysis

**Figure 1:**
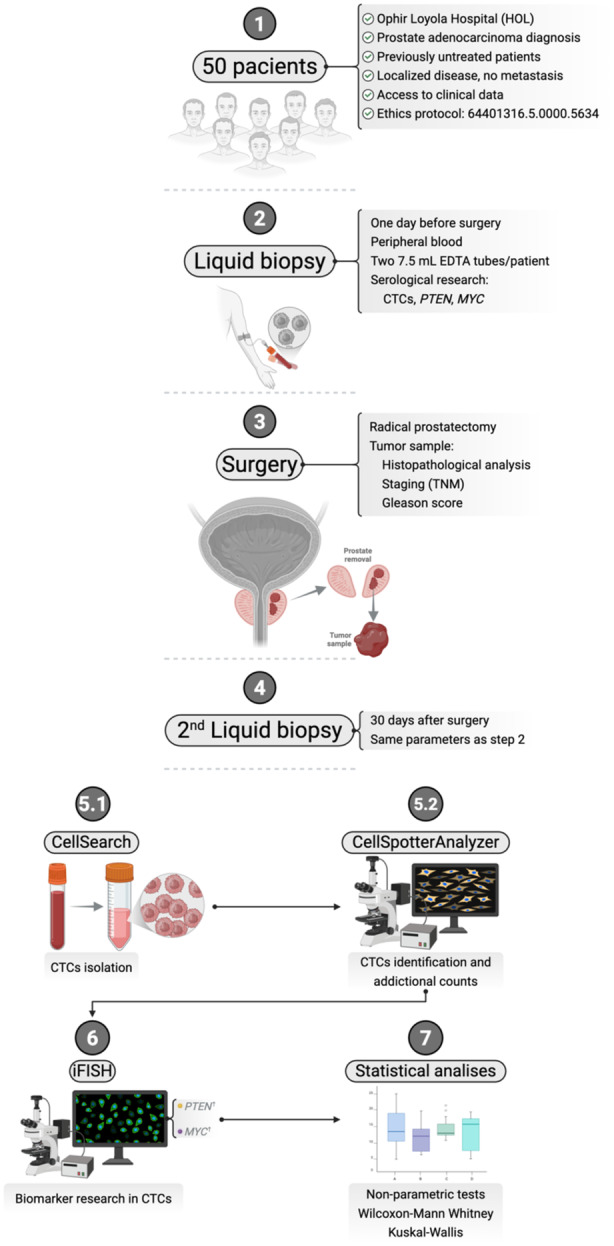
Methodological workflow used in the present study, from sample selection and collection to final analysis.

All statistical analyses were performed using RStudio version 4.4.1 [31] using dplyr and tidyr (data manipulation), ggplot2 (visualization), and tidyverse packages. Normality was assessed by the Shapiro–Wilk test; when not met, nonparametric tests were applied. Pairwise comparisons used the Wilcoxon rank‐sum (Mann–Whitney *U*) test, and three or more groups were analyzed with the Kruskal–Wallis test. Significance was set at *p* ≤ 0.05. The methodological summary of the study is presented schematically in Figure 1.

## 3 Results

In the present study, a total of 50 patients diagnosed with prostate adenocarcinoma were included. Table 1 provides a detailed analysis of the clinicopathological data of the study participants. Regarding age, the median was 64 years, with a range from 53 to 73 years. As for prostate‐specific antigen levels, preoperative data showed a mean PSA value of 10.7 ng/mL. Postoperatively, most patients exhibited undetectable PSA levels: 68% of the evaluated individuals had values below the detection limit (PSA < 0.1 ng/mL), with a median postoperative PSA level of 0.06 ng/mL. Concerning the Gleason score, approximately 80% of the patients had a score of 7, indicating a predominance of intermediate‐grade tumors in the studied group.

**Table 1:** Clinicopathological characteristics of the prostate cancer patients.

Variables	*N* = 50
Average age of diagnosis ± standard deviation	64.89 ± 6.4
Mean preoperative PSA ± Standard Deviation	10.7 ± 5.43
Gleason score	
≤ 6 (%)	5 (10)
7 (%)	40 (80)
≥ 8 (%)	5(10)
ISUP *N* (%)	
1	4 (8)
2	23 (46)
3	18 (36)
4	2 (4)
5	3 (6)
Tumor Node Metastasi (TNM) staging	
pT2	27
pT3	21
Seminal vesicle invasion *N* (%)	10 (20.83)
Presence of lymph node infiltration *N* (%)	1 (2.08)
Distant metástases	0 (0)
Mean postoperative PSA ± standard deviation	0.5 ± 1.596
< 0.1 (%)	34 (68)

Additional relevant findings include classification according to the ISUP grading system, in which 23 patients (46% of the total sample) were classified as ISUP grade 2, and 18 patients (36%) as ISUP grade 3. In terms of tumor staging, the majority of patients were classified as T2, indicating that the malignancy was confined to the prostate gland.

The analysis of parameters assessed both before and after radical prostatectomy revealed statistically significant results. In the evaluation of CTCs, preoperative counts ranged from 3 to 5 CTCs per 7.5 mL of blood. Following surgery, this count decreased to a range of 0 to 4 CTCs per 7.5 mL.

**Figure 2:**
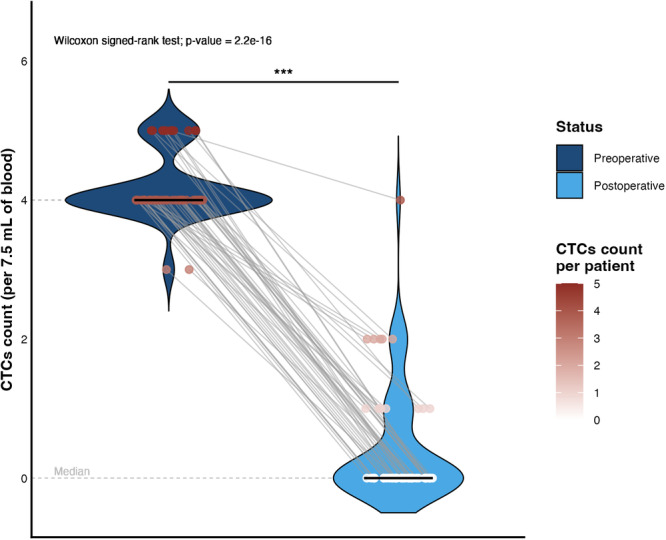
Comparison of circulating tumor cell counts in the peripheral blood of patients in the preoperative and postoperative periods. The distribution of CTC counts is represented by violin plots, with width proportional to the sampling density at each point on the *y*‐axis. The color intensity of the points indicates the absolute value of their respective counts (scale on the right). The gray lines connect the paired values for each patient, allowing visualization of the intra‐individual variation between the two clinical time points of radical prostectomy. The central horizontal black bars indicate the medians for each group. Note that after surgical resection, there was a substantial decrease in CTC counts, evidenced by strong statistical significance, reinforcing its potential as a dynamic biomarker for monitoring treatment response.

Notably, in 88% of patients, postoperative CTC levels declined to zero, reflecting a high PSA conversion rate. This finding was supported by a statistically significant difference observed between pre and postoperative values, as determined by the Wilcoxon signed‐rank test (*p* = 2.2 × 10⁻¹⁶). Figure 2 provides a clear and illustrative depiction of the marked reduction in CTC levels, which serves as a relevant indicator of tumor burden and treatment response in patients with PCa.

**Figure 3:**
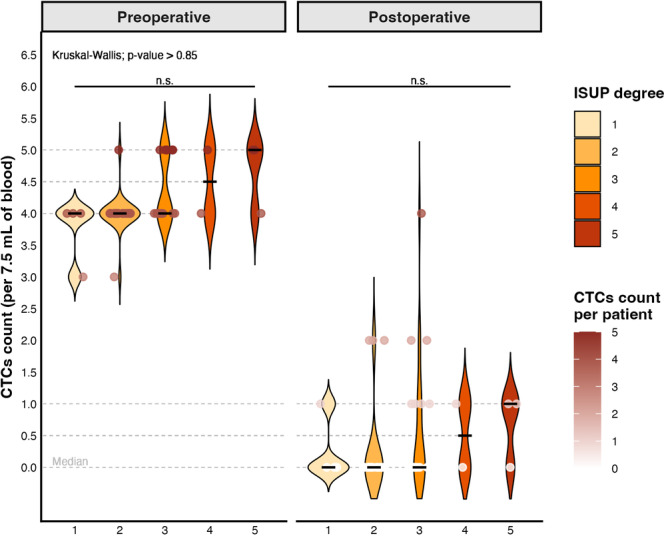
Distribution of CTCs by ISUP histological grade in the preoperative and postoperative periods. Tumor cell counts are represented by violin plots stratified by ISUP score, with the dots indicating absolute values for each patient and the horizontal bars representing medians. In the preoperative period, an increasing trend in CTC counts was observed as ISUP grade increased, with high medians in groups 4 and 5. However, this trend did not reach statistical significance. In the postoperative period, there was a clear decrease in CTC counts in all ISUP groups, with a concentration of values close to zero. Nevertheless, no statistically significant differences were observed between the groups after surgery, suggesting that the variation in response to surgical treatment, in terms of CTC reduction, was not dependent on ISUP histological grade but rather represented a beneficial clinical outcome. n.s., nonsignificance.

Analysis of the ISUP (Figure 3) grading system revealed that, among the 50 patients evaluated, 12 had an ISUP score of 2 or higher, indicating greater disease aggressiveness. Notably, these 12 patients were the same individuals who failed to achieve complete CTC clearance postoperatively. However, when investigating a potential statistical association between ISUP scores and CTC counts, no significant differences were observed either in the preoperative period (Kruskal–Wallis test; *p* = 0.85) or postoperatively (Kruskal–Wallis test; *p* = 0.21).

Despite the lack of statistical significance, the clinical relevance of this finding should not be overlooked, as illustrated in Figure 3. The results suggest that, although no direct and statistically significant correlation was identified between ISUP grade and CTC count, the persistence of elevated CTC levels in patients with higher ISUP scores may reflect a more aggressive disease phenotype, which warrants consideration in clinical decision‐making.

**Figure 4:**
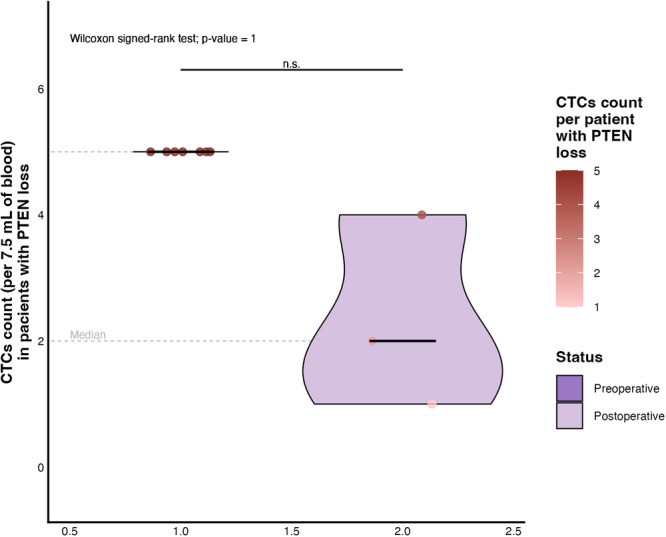
CTC count before and after radical prostatectomy in patients with *PTEN* gene deletion. The median CTC count differed between the two clinical time points, but no statistically significant difference was observed. The lower dashed line highlights the accumulation of patients without detectable CTCs after surgery. Despite the visual reduction in postoperative counts in some individuals, the small sample size may limit the statistical power of the analysis. Deletion of *PTEN*, a tumor suppressor gene involved in cell growth control and regulation of the *PI3K*/*AKT* pathway, has been associated with a more aggressive tumor phenotype. However, in this subset, the data suggest that the circulating CTC load did not vary significantly in response to surgical intervention, perhaps due to the initial tumor stage. n.s., nonsignificance.

Regarding the analysis of the *PTEN* gene, seven patients exhibited *PTEN* deletion prior to surgery. All of these individuals presented with a CTC count of 5 cells per 7.5 mL of blood. Following radical prostatectomy, only three of these patients retained *PTEN* deletion, and their CTC counts ranged from 1 to 4 cells. Although no statistically significant difference was observed (Mann–Whitney test; *p* = 1, the clinical relevance of this finding remains noteworthy, as illustrated in Figure 4.

*PTEN* loss was identified in seven patients, whose Gleason scores ranged from 7 to 9. In terms of ISUP grading, 71% of these patients were classified as ISUP grade 3, 14% as ISUP grade 5, and 14% as ISUP grade 2.

Analysis of pathological staging revealed that *PTEN* deletion occurred more frequently in patients classified as pT3aN0 and pT3bN0, while it was observed in only one patient with stage pT2N0. These findings indicate that *PTEN* loss is associated with more unfavorable pathological features (see Supporting Information: Table S1, for details).

**Figure 5:**
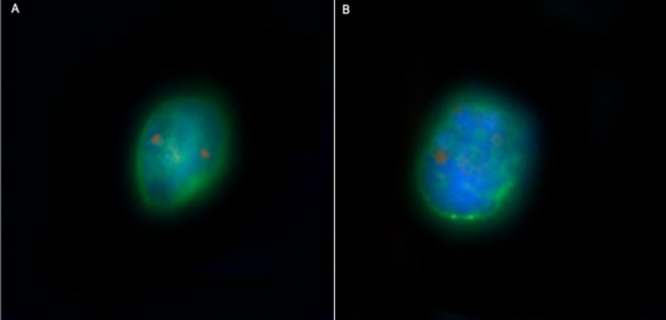
Detection of *PTEN* Deletion in CTCs by *SE‐iFISH*. (Panel A) shows a normal representation of the *PTEN* gene. (Panel B) depicts a homozygous deletion of the *PTEN* gene, evidenced by the presence of a single orange signal in a CTC (CK + /CD45 − /DAPI + /*PTEN*) identified using the SE‐iFISH platform. Magnification, ×400. DAPI, blue; *PTEN*, orange; CK, green; CD45, red (not expressed due to the tumor cell phenotype).

Figure 5 demonstrates the findings of the fluorescence in situ hybridization. *MYC* amplification was identified in a single patient, who presented with a Gleason score of 7, ISUP grade 2, pathological stage pT2N0, and concomitant *PTEN* deletion.

## 4 Discussion

PCa is a heterogeneous disease that manifests, even in its early stages, through diverse molecular and clinicopathological characteristics that directly influence prognosis and available therapeutic options [32]. Key prognostic factors include TNM staging, which evaluates tumor extent, lymph node infiltration, and metastatic spread; the Gleason score, which classifies neoplastic cell aggressiveness; and PSA levels, serving as detection and monitoring markers [32]. Furthermore, specific genetic mutations may impact tumor behavior and consequently therapeutic decisions [33]. This study enrolled intermediate‐ and high‐risk patients with a surgical indication for initial treatment, as shown in Table 1. While prostatectomy may cure many patients, approximately 30% experience biochemical recurrence, evidenced by postoperative PSA elevation [34]. Thus, refining clinical assessment models to distinguish men at high risk of early PCa mortality is critical, enabling aggressive salvage therapies while safely monitoring low‐risk patients.

LB studies emerge as promising tools for identifying poor‐prognosis patients, offering noninvasive disease monitoring and longitudinal detection of clinically relevant genetic alterations [35]. Substantial evidence supports CTC utility in metastatic disease evaluation [36]. However, clinical applicability in localized disease remains controversial, warranting further investigation to elucidate diagnostic and prognostic potential [37]. Our results demonstrate that all localized PCa patients exhibited baseline CTC levels of 3–5 cells pretreatment. Their presence even in early‐stage disease suggests early hematogenous dissemination [38, 39]. The 88% postoperative CTC clearance rate indicates primary tumor removal may eliminate circulating cells, potentially reflecting treatment efficacy and providing therapeutic response monitoring. Persistent postoperative CTCs in some patients raise concerns about metastatic risk, necessitating rigorous surveillance or adjuvant therapy intensification.

In the literature, there is still a limited number of studies investigating the role of CTCs in early‐stage PCa. The available results remain controversial: while some studies suggest that the presence of CTCs may be associated with a higher risk of disease progression [40], others have failed to establish a consistent correlation [41]. These findings underscore the need for studies with larger patient cohorts to validate the clinical utility and applicability of this approach. Furthermore, the standardization of CTC collection and analysis protocols is essential to ensure data comparability across studies.

On the other hand, some studies show results similar to those explored in the present work. Researchers examined CTCs in PCa patients undergoing cytoreductive radical prostatectomy and found no significant difference in clinicopathological variables, operative parameters, or long‐term oncological outcomes between groups with and without increased perioperative CTCs. However, the level of CTCs decreased significantly after surgery, and they mention that perioperative cytoreduction may represent a safe procedure for the treatment of patients with metastatic hormone‐sensitive prostate cancer (MHSC); however, they indicate that trials should be conducted in larger groups of patients [42].

Another study highlighted that variations in CTC levels are directly related to both the method used for their detection and the timing of sample collection. The authors observed that the mere presence of CTCs, regardless of quantification, constituted an autonomous risk factor associated with PCa recurrence after prostatectomy (*p* < 0.001). They further suggest that the detection of CTCs by the ISET® method in the preoperative period may act as a promising predictive marker for recurrence in patients with nonmetastatic PCa [43].

In the context of biomarkers, one study showed that increased MYC expression is correlated with progression and recurrence after prostatectomy; MYC is frequently cited as an early alteration associated with aggressive PCa behavior. However, this study does not show a “postoperative reduction” of MYC, since MYC is evaluated in the removed tumor tissue (not in LB as done in our study) and its presence/alteration is linked to tumor behavior [44].

A review published in 2019 by Broncy and colleagues focusing on PCa and CTCs in localized disease evaluated 11 studies and confirmed the scarcity of research in this setting [45]. The review highlighted a consistent trend suggesting that CTC counts correlate with pathological stage and may have prognostic and predictive implications in early‐stage PCa. Additionally, several studies have reported significant correlations between CTC numbers and patient survival and/or disease recurrence after treatment [46, 47].

In the present analysis, among the 50 patients evaluated, 12 presented an ISUP score of 2 or higher, reflecting greater tumor aggressiveness according to the updated histological grading system of the ISUP. Notably, the same 12 patients failed to achieve postoperative CTC clearance, suggesting a potential association between histological grade and the persistence of hematogenous dissemination.

Despite this consistent clinical observation, statistical analysis did not reveal a significant association between ISUP scores and CTC counts, either in the preoperative period (*p* = 0.85) or postoperatively (*p* = 0.21). These findings indicate that, within the evaluated sample, it was not possible to establish a robust statistical correlation between tumor histopathology and CTC dynamics. Nevertheless, the absence of statistical significance does not invalidate the clinical relevance of the observed pattern, especially considering the small number of cases.

With respect to *PTEN* assessment, seven cases of *PTEN* deletion were identified. It is well established that the loss of *PTEN* function leads to dysregulated activation of the *PI3K*/*AKT* signaling pathway, promoting tumor cell proliferation and growth. Previous studies have shown that *PTEN* deletion is associated with worse prognosis, higher risk of disease progression, and lower overall survival [48]. Data from The Cancer Genome Atlas have demonstrated that *PTEN* gene deletion occurs in approximately 40% of advanced PCa cases and is strongly associated with tumor progression, castration resistance, and reduced overall survival.

A study conducted by [13] involving patients from Spain and Latin America, *PTEN* deletion was detected in approximately 28% of patients with localized PCa and 54% of those with advanced disease. Furthermore, the combined presence of *PTEN* deletion and *MYC* amplification in CTCs was associated with nearly a twofold increased risk of biochemical recurrence following radical prostatectomy, reinforcing the prognostic and risk stratification potential of these biomarkers. These findings are consistent with the results of the present study, indicating that *PTEN* alterations may serve as early markers of tumor persistence even after surgical treatment.

Additional insight was provided by [39], who analyzed CTCs from patients with castration‐resistant PCa and demonstrated that *MYC* amplification in CTCs was associated with increased bone metastasis and reduced response to second‐line hormonal therapy. The study, which included patients from diverse ethnic backgrounds, including Latin Americans, also emphasized that the simultaneous detection of *MYC* and *PTEN* alterations significantly enhances the predictive value for disease progression. Incorporating these biomarkers into routine clinical practice could benefit the studied Amazonian population, particularly by enabling more accurate risk stratification and personalized therapeutic interventions [39]. These findings suggest that *PTEN* status assessment may be a valuable tool in risk stratification and in guiding more precise therapeutic strategies in the management of PCa.

It is known that PCa exhibits varying degrees of DNA copy number alterations. Indolent tumors and those with low Gleason scores tend to display fewer alterations, whereas more aggressive primary and metastatic tumors often exhibit widespread genomic copy number changes [49, 50]. Among these alterations, *MYC* gene amplification stands out as one of the earliest molecular events identified in prostate tumors, including those in localized disease. Studies have shown that *MYC* amplification can occur in early stages of tumorigenesis and is associated with increased genomic instability and tumor progression potential, even in cases with seemingly less aggressive clinical features [51].

In the present study, *MYC* gene amplification was identified in only one patient, who presented with a Gleason score of 7 (ISUP 2), pathological stage pT2N0, and concurrent *PTEN* deletion. The low frequency observed may be attributed to the reduced circulating tumor burden in the analyzed cohort, particularly when compared to studies conducted in metastatic disease contexts. Moreover, most studies investigating *MYC* amplification in localized PCa are based on tumor tissue analyses, which may offer greater sensitivity for detecting such alterations [52]. Consequently, the limitations of LB‐based methodology may have contributed to the low detection rate in this study.

## 5 Conclusions

The findings of this study reinforce the translational potential of CTCs as minimally invasive biomarkers for risk stratification in patients with localized PCa, expanding the current understanding of their prognostic relevance in this disease stage. The detection of CTCs prior to radical prostatectomy, even in early‐stage disease, coupled with the significant negative conversion following treatment, supports their prognostic value and potential applicability in therapeutic response monitoring. CTCs demonstrate promise as an additional tool for prognostic and predictive evaluation, particularly in determining the need for adjuvant therapy or therapeutic intensification through multimodal approaches.

**Figure 6:**
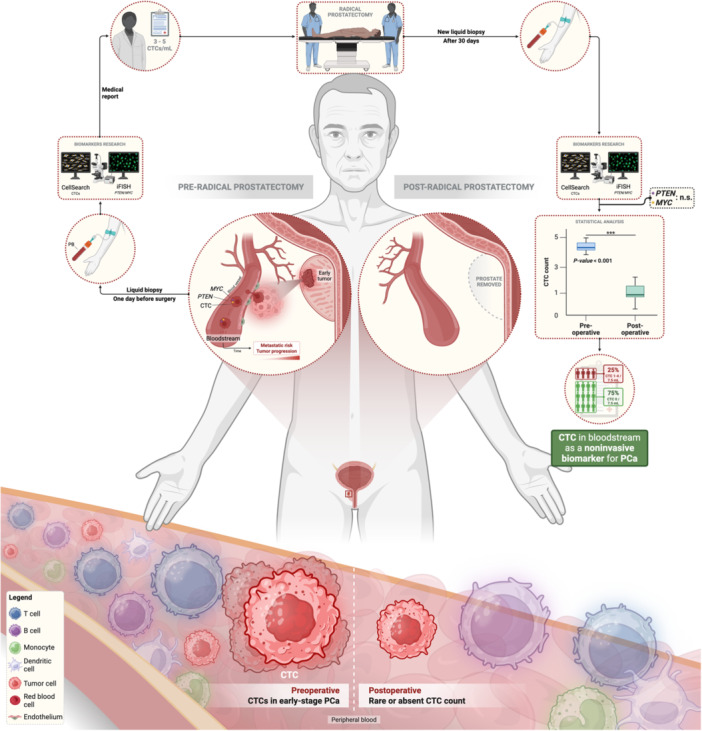
Schematic representation of the use of circulating tumor cells as noninvasive biomarkers for early screening and monitoring of prostate cancer (PCa). Peripheral blood samples were collected from patients one day before and 30 days after radical prostatectomy, with subsequent analysis by cytometry (CellSearch®) for CTC quantification and fluorescence in situ hybridization (iFISH) for detection of genetic alterations in the *PTEN* and *MYC* genes. The presence of CTCs was observed preoperatively, which may increase the risk of metastasis and tumor progression. Postoperatively, there was a significant reduction in the number of CTCs, while *PTEN* and *MYC* showed no statistically significant difference between the two clinical time points. The image also illustrates the composition of peripheral blood, highlighting the greater presence of CTCs preoperatively compared to the rare or absent presence postoperatively. These findings reinforce the potential of CTCs as noninvasive biomarkers for early diagnosis and assessment of surgical response in PCa.

Furthermore, their use may represent a valuable molecular‐based risk stratification strategy, aiding in the distinction between indolent and aggressive tumors. However, further studies with larger population samples are needed to validate the clinical utility of CTCs in localized PCa. Figure 6 below summarizes the study presented here, from initial patient screening to final clinical findings.

## Author Contributions

**Juliana Ramos Chaves:** study concept and design, acquisition of data, drafting of the manuscript, statistical analysis. **Rommel Mario Rodriguez Burbano:** study concept and design, acquisition of data, obtaining funding, administrative, technical, or material support. **Amanda de Nazaré Cohen‐Paes:** analysis and interpretation of data, drafting of the manuscript, critical revision of the manuscript for important intellectual content, statistical analysis. **Diego Di Felipe Ávila Alcântara:** acquisition of data, drafting of the manuscript. **Ana Paula Borges de Souza:** critical revision of the manuscript for important intellectual content. **Maria Janete Nahum Gomes:** analysis and interpretation of data. **Sérgio Augusto Antunes Ramos:** analysis and interpretation of data, critical revision of the manuscript for important intellectual content, statistical analysis. **André Salim Khayat:** study concept and design, acquisition of data, drafting of the manuscript, drafting of the manuscript, critical revision of the manuscript for important intellectual content, statistical analysis, supervision. Juliana Ramos Chaves had full access to all the data in the study and takes responsibility for the integrity of the data and the accuracy of the data analysis.

## Supporting information


**Table S1:** Clinicopathological data of patients with deleted *PTEN*.

## Data Availability

The authors have nothing to report.
