# Supplementary material for: Dynamic Profiling of Circulating Tumor Cells and MYC/PTEN Alterations in Prostate Cancer Patients Undergoing Radical Prostatectomy in Amazon Population
Source: Mol Carcinog. 2026 Mar 6;65(6):665–76. doi: 10.1002/mc.70104 (PMC13178411; doi:10.1002/mc.70104)
Supplement: Supplementary file 1 — Table S1: Clinicopathological data of patients with deleted PTEN. [file MC-65-665-s001.docx]

***Supplementary Material***

**Table S1.** Clinicopathological data of patients with deleted *PTEN*.

| **Pacient** | **Age** | **PSA (ng/mL)** | **ISUP** | **Gleason *score*** | **CTC Preoperative** | ***PTEN* *status*** | **TNM Classification** |
| --- | --- | --- | --- | --- | --- | --- | --- |
| 1 | 61 | 12,02 | 5 | 4+5 | 5 | Pten Loss | T3aNxM0 |
| 2 | 54 | 23,63 | 3 | 4+3 | 5 | Pten Loss | T3bNxM0 |
| 3 | 73 | 14,71 | 3 | 4+3 | 5 | Pten Loss | T3bN1M0 |
| 4 | 72 | 6,77 | 3 | 4+3 | 5 | Pten Loss | T3aNxM0 |
| 5 | 67 | 6,04 | 3 | 4+3 | 5 | Pten Loss | T3bNXM0 |
| 6 | 67 | 6,1 | 3 | 4+3 | 5 | Pten Loss | T3bNXM0 |
| 7 | 60 | 8,44 | 2 | 3+4 | 5 | Pten Loss | T2pNxM0 |
